# Flexible Polymer Electrodes for Stable Prosthetic Visual Perception in Mice

**DOI:** 10.1002/adhm.202304169

**Published:** 2024-03-03

**Authors:** Corinne Orlemann, Christian Boehler, Roxana N. Kooijmans, Bingshuo Li, Maria Asplund, Pieter R. Roelfsema

**Affiliations:** ^1^ Department of Vision and Cognition Netherlands Institute for Neuroscience Royal Netherlands Academy of Arts and Sciences Amsterdam 1105 BA The Netherlands; ^2^ Department of Microsystems Engineering (IMTEK) University of Freiburg 79110 Freiburg Germany; ^3^ BrainLinks‐BrainTools Center University of Freiburg 79110 Freiburg Germany; ^4^ Institute for Neuroscience and Medicine (INM‐1) Forschungszentrum Jülich 52428 Jülich Germany; ^5^ Department of Microtechnology and Nanoscience Chalmers University of Technology Gothenburg 412 96 Sweden; ^6^ Laboratory of Visual Brain Therapy Sorbonne Université Institut National de la Santé et de la Recherche Médicale Centre National de la Recherche Scientifique Institut de la Vision Paris F‐75012 France; ^7^ Department of Integrative Neurophysiology Centre for Neurogenomics and Cognitive Research VU University Amsterdam 1081 HV The Netherlands; ^8^ Department of Neurosurgery Amsterdam University Medical Center University of Amsterdam Amsterdam 1105 AZ The Netherlands

**Keywords:** bioengineering, cortical implant, microelectrodes, vision, visual prosthesis

## Abstract

Brain interfaces that can stimulate neurons, cause minimal damage, and work for a long time will be central for future neuroprosthetics. Here, the long‐term performance of highly flexible, thin polyimide shanks with several small (<15 µm) electrodes during electrical microstimulation of the visual cortex, is reported. The electrodes exhibit a remarkable stability when several billions of electrical pulses are applied in vitro. When the devices are implanted in the primary visual cortex (area V1) of mice and the animals are trained to detect electrical microstimulation, it is found that the perceptual thresholds are 2–20 microamperes (µA), which is far below the maximal currents that the electrodes can withstand. The long‐term functionality of the devices in vivo is excellent, with stable performance for up to more than a year and little damage to the brain tissue. These results demonstrate the potential of thin floating electrodes for the long‐term restoration of lost sensory functions.

## Graphical abstract

Flexible polymer‐based electrodes of minimal size (<15 um^2^) exhibit exceptional stability, enduring billions of pulses in vitro. In mice, microstimulation of the visual cortex elicits perception at current amplitudes as low as 2 µA and electrodes exhibit sustained functionality up to 55 weeks. Prolonged implantation triggers limited immune responses, highlighting advantages of flexible electrodes for both performance and tissue integration.

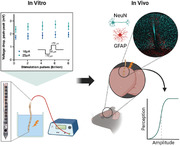

## 1 Introduction

Recent studies provide evidence that patterned electrical stimulation of the central nervous system through implanted electrodes can generate complex sensory percepts, thereby positioning neurotechnology as a promising therapeutic method for restoring lost sensory functions.^[^ ^1^, ^2^, ^3^, ^4^, ^5^, ^6^ ^]^ It has been well established that the stimulation of neurons in the visual cortex evokes an artificial perception of light called a “phosphene”.^[^ ^7^, ^8^, ^9^, ^10^ ^]^ We recently demonstrated that the simultaneous, patterned stimulation of several electrodes in area V1 o monkeys, using multiple silicon‐based Utah arrays, elicits shape perception.^[^ ^11^ ^]^ This previous study provided proof‐of‐principle that electrical stimulation of the visual cortex permits prosthetic vision of more complex shapes. However, the creation of a clinically applicable visual prosthetic system based on Utah arrays remains challenging because the functionality of Utah arrays degrades over time.^[^ ^12^, ^13^, ^14^, ^15^, ^16^, ^17^ ^]^

Deterioration of the interface can be caused by insufficient biostability of the materials, electrochemical degradation of electrodes, and the brain's foreign body response to the implant.^[^ ^12^, ^18^ ^]^ The silicon‐based electrode arrays are rigid and do not follow the movements of the brain during respiration and the heart‐beat, resulting in increased gliosis and elevated stimulation thresholds.^[^ ^19^, ^20^ ^]^ Gliosis is expected to be pronounced when large numbers of arrays are needed, as is required to cover the entire visual field.^[^ ^11^, ^13^ ^]^ Ultra‐flexible, polymer‐based shanks may offer a solution because they follow the movements of the brain tissue and their small dimensions ensure minimal disruption of the tissue perfusion. This technology may also provide a feasible approach for the coverage of a brain region with high numbers of electrodes without compromising tissue integrity. Previous studies have shown that flexible probes trigger little glial scarring and allow stable recording quality for extended periods.^[^ ^21^, ^22^, ^23^, ^24^ ^]^ We therefore hypothesized that the reduced foreign body response elicited by small, flexible electrodes would also be beneficial for cortical stimulation because the stimulation threshold might remain low, increasing the likelihood they can be used for extended periods^[^ ^25^ ^]^ and a recent study provided evidence for stable performance of flexible polymer‐based microelectrodes in the somatosensory cortex of mice.^[^ ^26^ ^]^

The currents that are used to activate neurons with stiff silicon electrodes are usually in the range of tens of µA,^[^ ^11^, ^27^, ^28^ ^]^ which is difficult to achieve with smaller electrodes on thin flexible shafts. As the metallization is based on thin‐film technologies, minor stimulation induced charges might cause degradation of the electrode material. Indeed, delamination of thin‐film electrodes has been frequently reported;^[^ ^29^ ^]^ although, a recent study demonstrated improved stability under stimulation in the peripheral nervous system (Čvančara et al., 2023). Visual prosthetic applications are especially demanding because exceptionally small electrodes are needed to achieve a good resolution of prosthetic vision; while, at the same time electrodes need to operate close to continuously provide real‐time visual information to the patient. On the one hand, it is unknown whether sufficient charge injection capacity can be achieved for the system to operate stably over time. On the other hand, the reduced gliosis response caused by highly flexible electrodes is expected to decrease the distance between the neurons and the electrodes, which could reduce the current necessary to activate them.^[^ ^30^, ^31^ ^]^ Our study examined whether the charge injection capacity of electrodes on flexible shanks suffices for the induction of phosphenes over long usage periods. We report that polymer‐based thin‐film electrode arrays successfully elicit perceptions in mouse V1, at an average current strength of 8 µA, which is well below the maximal currents that these electrodes can sustain. Importantly, we also show that this functionality is retained even over long implantation times, up to more than 1 year.

## 2 Results

To maximize the stimulation current that could be passed through the small electrodes (15 × 15 µm^2^), we used sputtered iridium oxide (SIROF) as electrode material and added a coating of poly(3,4‐ethylene‐dioxythiophene)/polystyrene sulfonate (PEDOT/PSS), which acted as capacitive/pseudo‐capacitive charge injection layer. This way, we reached a maximum charge injection of 3.3 mC cm^−2^; so that, we could use current amplitudes up to 36 µA (192 nA µm^−2^) without risking electrolysis at the electrode surface.^[^ ^32^ ^]^

**Figure 1:**
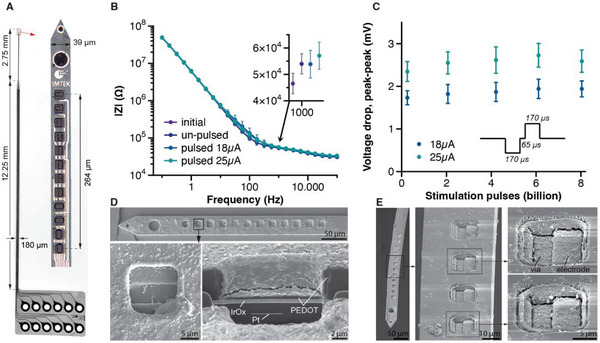
Flexible probe characteristics. A) Photograph of the multi‐layer probe with overall dimensions and a close‐up showing the distribution of the electrode sites at the tip of the probe. B) Impedance spectrum of an unused probe (purple) and measured after exposing the individual electrode sites to biphasic stimulation over more than 10 billion stimulation pulses at an amplitude of 18 µA (blue) and 25 µA (green). The un‐pulsed control group is shown as dark blue circles; solid lines represent the mean (*n* = 4). The inset shows the impedance at 1 kHz for all groups (mean ± SD). C) Voltage‐drop at the electrodes during biphasic pulsing in vitro over a period of 16 weeks with more than 10 billion stimulation pulses. The full dataset is shown in Figure S1, Supporting Information. D) Scanning electron microscopy images of the probe exposed to the pulsing test in (B). Higher‐magnification view to the left shows a representative electrode site (here, pulsed with 25 µA) in top view. The right image shows a cross‐sectional view of an electrode site prepared with focused ion beam detailing the metallization layers (Pt, IrO*x*) and the PEDOT coating. E) Scanning electron microscopy images of a probe explanted after 55 weeks in vivo, with close‐up images showing the PEDOT coated electrode sites after repeated stimulation in vivo (see Table S2, Supporting Information). A small crack along the edge of the electrode bottom, marking the transition from the horizontal to the vertical plane, is visible for both stimulated and non‐stimulated electrode sites, likely resulting from sample preparation for SEM imaging.

Electrical stimulation may change the electrode properties, including the impedance, if residual charges accumulate over time.^[^ ^33^ ^]^ In these situations, currents that initially are safe may later result in a larger voltage drop across the electrode, causing electrolysis and further degradation. Thin‐film electrodes are particularly vulnerable to these irreversible electrochemical reactions because of their high surface‐to‐volume ratio.^[^ ^34^ ^]^ To ensure that stimulation would not jeopardize electrode integrity; we therefore extensively tested the resilience of the electrodes to continuous pulsing at 18 and 25 µA (1.7 and 2.3 mC cm^−2^, corresponding to 50% and 75% of the maximum charge injection) in saline, at a frequency of 1 kHz for a test period of 16 weeks reaching a total of more than 10 billion pulses. We compared the electrochemical impedance before and after the 10 billion pulses and found that the difference was barely noticeable (**Figure** **1**B). The impedance at 1 kHz was 46.5 ± 3.9 kΩ (mean ± SD) before and 53.9 ± 5.2 and 57.1 ± 5.2 kΩ after pulses of 18 and 25 µA, respectively (*p* > 0.79 and *p* > 0.37). The voltage drop over the electrode increased by only 13% over the entire 16 weeks of continuous pulsing (from 2.3 ± 0.2 V to 2.5 ± 0.3 V, Figure 1C). To further investigate if any electrode degradation took place, we used focused ion beam (FIB) with scanning electron microscopy (SEM) to investigate the structural integrity of the electrodes after pulsing (Figure 1D). The layers of the electrode were intact, including the contact with the connection lines and the SIROF and PEDOT layers. In some cases, we observed cracks in the PEDOT layer, typically along the rim of the electrode (Figure 1E). Electrodeposited PEDOT electrodes incorporated electrolyte during their synthesis and shrank substantially upon drying. Hence, it is not unexpected that cracks formed during the preprocessing steps for SEM when the film was fully dehydrated under vacuum; although, we cannot exclude that cracks were caused by the electrical stimulation. Importantly, the film remained stably adherent to the conducting SIROF support, in spite of the cracks, and impedance spectroscopy indicated that the cracks did not impair electrode performance. The stability of electrodes depended on the design of the stimulator,^[^ ^32^ ^]^ and we replicated our results with a second stimulator, the Blackrock Cerestim (Figure S2, Supporting Information), confirming that the electrodes tolerated stimulation well. We conclude that the electrodes permitted electrical stimulation over extended time periods and with an overall high amount of charge (42.5 C over 10 B pulses, 226 mC µm^−2^); so that, we could proceed to test electrical stimulation in mice.

### 2.1 Microstimulation Evokes Sensory Percepts With a Low Perceptual Threshold

**Figure 2:**
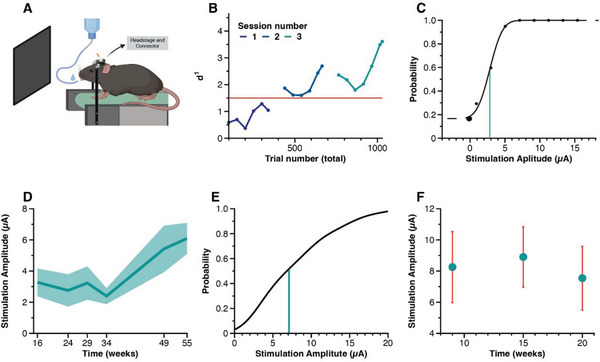
In vivo stimulation and detection threshold. A) Illustration of the behavioral set‐up. The mice were head‐fixed on a treadmill in front of a blank monitor (image created with Biorender.com). We applied electrical stimulation via a connector and trained the mice to perform a licking response to the stimulation current, which was rewarded with water. B) Detection performance of mouse 1 to stimulation of electrode 6 during a go/no‐go detection task over three sessions. Performance was assessed as d‐prime measured in epochs of 100 trials, using a moving window of 50 trials. The d‐prime starts at zero for untrained mice. The red line marks the accuracy at which we considered the mouse to be proficient in the task. C) Example psychometric function of electrode 6 of mouse 1. The *y*‐axis shows lick probability as function of stimulation strength (*x*‐axis). The vertical green line marks the perception threshold (*θ* = 2.9 µA), defined as the stimulation amplitude necessary to achieve 60% accuracy. D) Detection thresholds in mouse 1 over 55 weeks (mean ± SD, *N* = 7 electrodes). E) Cumulative distribution of detection thresholds of 23 electrodes across the five probes. The vertical line depicts the median detection threshold, which was 7.1 µA (1.2 nC per phase). F) To gain insight into the time‐course of the stimulation thresholds, all measurements were assigned to one of three epochs: early (mean = 9 weeks), intermediate (mean = 15 weeks), and late (mean = 20 weeks). Thresholds are presented as mean ± SEM. We did not observe a clear trend in the current threshold over time.

We tested the functionality of the probes by implanting them in area V1 of five mice (1 probe per mouse). We trained the mice to report microstimulation (initially 25 µA with 4.25 nC per phase, biphasic pulses at 300 Hz for 240 ms, see Experimental Section) by licking a spout (**Figure** **2**A). The mice learned to respond to the microstimulation after a median of seven sessions. The behavior of one example mouse across three training sessions is shown in Figure 2B. This mouse reached a behavioral d‐prime of 2 in the second session and it improved further in later sessions. Once the mice had learnt the task, we determined the threshold current that gave rise to 60% hits by varying the current strength across trials. The threshold current for an example electrode was 2.9 µA (0.49 nC per phase, Figure 2C, same mouse as in Figure 2B), and the average threshold for this mouse across seven electrodes was 3.3 ± 0.9 µA (0.56 ± 0.15 nC per phase; mean ± SD; range, 2.5 to 5.1 µA; see Table S1, Supporting Information, for the data of all mice). In this mouse, we were able to monitor the thresholds of the same seven electrodes across 55 weeks and observed a slight increase to an average of 6.1 ± 1 µA (1.04 ± 0.17 nC per phase; range, from 4.5 to 7.8 µA) (Figure 2D) (Kruskal Wallis test, *χ* ^2^(5) = 25.9; *p* < 0.0001).

Overall, we determined the detection thresholds for 23 electrodes implanted in five mice and obtained an average detection threshold of 8.3 ± 5.6 µA (1.41 ± 0.95 nC per phase; range, from 1.8 to 20.2 µA). For most of the electrodes, we obtained a single measurement of the current threshold. To gain further insight of the development of current thresholds, we therefore binned the time‐points of the threshold measurements for the 23 electrodes into 3 epochs (time bins centered on 9, 15, and 20 weeks; Figure 2F). The average threshold was 8.3 ± 6.5 µA (1.4 ± 1.1 nC per phase) in the first epoch, 8.9 ± 5.5 µA (1.51 ± 0.93 nC per phase) in the second epoch, and 7.5 ± 5.4 µA (1.3 ± 0.9 nC per phase) in the last epoch. This analysis did not reveal a significant influence of the number of weeks since implantation on the current threshold (Kruskal Wallis test, *χ* ^2^(2) = 0.36; *p* = 0.84). We note however, that some of the electrodes lost their functionality during the experiment, for various reasons (Table S1 and other Supporting Information).

### 2.2 Probe Implantation Induced a Limited Glial Response

We examined the explanted probes to investigate their integrity using SEM and found that they were largely unchanged despite the long implantation times. In some of the explanted electrodes, we observed cracks that were similar to those observed during the in vitro analysis (Figure 1E, 55 weeks in vivo; same probe as in Figure 2B‐D). As was mentioned in the above, we do not know when these cracks formed. In addition to swelling of the film during the long implantation times followed by de‐swelling of the film upon explant preparation, these cracks could have been caused by the cleaning procedures that were used to remove the tissue. Similar cracks were observed for stimulated and non‐stimulated electrodes, supporting the assumption that the effect was unrelated to stimulation. Importantly, the cracks did not cause flaking of the film or other functional failure because it remained adherent to the underlying SIROF and they did not cause electrode dysfunction. Indeed, the probe in Figure 1E remained functional for 55 weeks in vivo. In short, the functionality of electrodes with and without such cracks was preserved.

**Figure 3:**
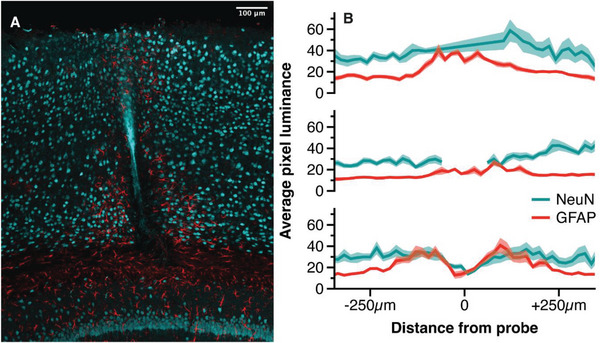
Immunohistochemistry of the probe lesion. Histological analysis of the tissue response to probes that had been implanted for 24 weeks in area V1 of the mouse visual cortex. A) Merged image of NeuN positive‐cells marking neuronal nuclei (blue) and GFAP‐positive cells marking astrocytes (red). Scale bar = 100 µm. B) Quantification of signal intensity (pixel luminance) of NeuN (blue) and GFAP (red) at superficial layers (top), along the shaft of the probe (middle), and at the tip of the probe (bottom). Each region of interest includes datapoints from ten horizontal lines taken from two animals. Shaded area marks SEM. Datapoints with an autofluorescence artefact (at the shaft of the probe) were removed.

We developed an ex vivo histological staining protocol to investigate the tissue response and integration of the probe (for details see Experimental Section). As expected, the implanted probes created a lesion with a width of ≈75 µm, which could be seen across the cortical depth (**Figure** **3**A,B). We inspected tissue responses at three depths: at the superficial layers (Figure 3B top), along the shaft of the probe (Figure 3B middle), and at the tip of the probe (Figure 3B bottom). To examine the neuroinflammatory response, we labeled the brain tissue for GFAP, marking for reactive astrocytes. We observed a low number of reactive astrocytes in the vicinity of the probe. Minimal reactivity was evident along the shaft of the probe (Figure 3B middle) and increased with depth reaching the highest level at the tip of the probe where reactive astrocytes occurred up to a distance of 150 µm from the probe (Figure 3B bottom). We examined neuronal density and neuronal loss around the implant by labeling the brain tissue for NeuN, which stains neuronal nuclei. We observed a reduction of neural density that was most pronounced at larger cortical depths. At the tip of the probe, the neuronal density was reduced up to a distance of 70 µm from the probe (Figure 3B bottom); although, neuronal nuclei were not completely absent from this region of cortex. Hence, neurons were present in the immediate vicinity of the implant, but, in lower numbers than in pristine tissue.

## 3 Discussion

Here, we examined whether flexible polymer‐based probes with small (15 × 15 µm^2^) electrodes can be used for the long‐term electrical stimulation of brain tissue. Most previous studies on electrical microstimulation have used silicon arrays and reported successful stimulation for several months and up to 4 years at current levels of tens to hundreds of µA.^[^ ^3^, ^6^, ^35^, ^36^, ^37^, ^38^, ^39^ ^]^ The median time to failure of the Utah arrays, one of the most used arrays made of silicon, is less than a year. There are several causes for these failures, including material and mechanical failures and foreign body responses that include gliosis and macrophage infiltration. The foreign body response can in part be mediated by the meninges and may cause cortical atrophy.^[^ ^12^, ^13^, ^40^ ^]^

Our usage of flexible polyimide probes was inspired by previous studies suggesting that the cross‐section and flexibility of the implanted neural probes plays an important role in long‐term stability. Thin polymer probes are highly flexible and displace little brain tissue during implantation, factors that contribute to their excellent long‐term recording quality.^[^ ^21^, ^24^, ^41^ ^]^ This recording quality is a useful feature, but a more important demand on electrodes for a sensory prosthesis is the long‐term application of currents that activate enough neurons to elicit perception. To our knowledge, only one other study has examined the long‐term perception of microstimulation via polymer‐based electrode arrays in the brain,^[^ ^26^ ^]^ as will be discussed below.

Micro‐stimulation using thin‐film metallization is known to challenge the electrode integrity.^[^ ^29^ ^]^ Here, we demonstrated that efficient and long‐term stable micro‐stimulation is possible. Our in vitro tests demonstrated stable electrode performance during ten billion pulses with an amplitude well above the thresholds for eliciting perception. Indeed, the higher amplitude of 25 µA (2.3 mC cm^−2^) tested long‐term in vitro was expected to bring the electrode to 2/3rds of the boundary of the water window, which was the current at which water electrolysis started to occur (3.2 mC cm^−2^). The applied charge density exceeded clinically reported thresholds for eliciting visual percepts (374 µC cm^−2^ in Fernández et al.^[^ ^3^ ^]^) by more than factor 6, illustrating the suitability of our electrodes for long‐term applications. Let us assume that the typical electrode is stimulated at 300 Hz in a prosthesis, and that these electrodes are active for an average of one in six images that are imposed on the visual brain, with an average usage of the device for 16 h per day. Under these assumptions, 10 billion pulses correspond to a lifetime of more than 8 years. In fact, the in vitro results are compatible with longer lifetimes because we did not obtain evidence for deterioration of electrode performance after this high number of pulses in vitro, corresponding to a total applied charge of more than 42 °C (226 mC µm^−2^). The number of pulses exceeded those reported by Lycke et al.,^[^ ^26^ ^]^ who tested 50 million pulses, corresponding to a total charge of 150 mC (332 µC µm^−2^). In contrast to Lycke et al. and the present results, Woeppel et al.^[^ ^17^ ^]^ reported considerable electrode damage after applying a total charge of only 130 µC (70 nC µm^−2^). While SEM imaging in the present study revealed small cracks in pulsed and explanted electrodes after stimulation in vivo, similar cracks were also observed previously with electrodes exclusively used for recording of neural activity in the mouse cortex over more than 25 weeks.^[^ ^21^ ^]^ This supports our hypothesis that these cracks are not caused by electrical stimulation but occur during the preparation of the sample for SEM imaging. Irrespective of their cause, our data supports that the formation of a crack has no immediately negative effect on electrode recording and stimulation performance.

It is difficult to predict in vivo performance based on in vitro stimulation because the circumstances in the brain may have both positive and negative impact on the electrode stability. On the one hand, the immune system can attack the implant and produce reactive agents that are not present in vitro.^[^ ^42^, ^43^ ^]^ On the other hand, experience from cochlear implants has shown that early predictions of electrode deterioration may not always substantiate in vivo because the environment can also provide protective influences.^[^ ^44^ ^]^ Further, in continuous in vitro pulsing experiments, residual polarization will accumulate, whereas the in vivo duty‐cycle leaves more leeway for electrodes to neutralize between pulse trains.^[^ ^32^ ^]^ Importantly, both in vitro and in vivo analysis reveal excellent stability of the electrodes and our results demonstrate that they withstand repeated charge injection without deterioration, even over the long timeframes that are relevant for visual prosthetics.

The factors that determine longevity in vivo include the foreign body response that may cause encapsulation. There are both acute and chronic triggers for an inflammation response. The implantation of probes causes acute damage to the brain tissue, which triggers a response of the immune system and also of repair mechanisms during a phase that lasts several weeks.^[^ ^16^ ^]^ This acute response is followed by a chronic inflammation response caused by the continuous presence of probes in the brain, which can lead to glial scarring and damage to the surrounding neurons.^[^ ^45^, ^46^, ^47^ ^]^ The encapsulation of the electrodes by glial cells impedes neuronal activation and thereby impairs the electrodes’ efficacy.^[^ ^12^, ^16^, ^48^ ^]^ These problems can be mitigated by improving the biocompatibility of the implant, and our results indicate that this improvement is compatible with current injection at a level necessary to elicit phosphenes across longer time periods.

We here analyzed the electrode performance in mice, which reported electrical microstimulation of the primary visual cortex by licking. In humans and monkeys, V1 stimulation elicits the perception of small dots of light, known as phosphenes. Phosphenes appear at the location in the visual field that corresponds to the electrode's position of the V1 map of visual space.^[^ ^11^, ^49^, ^50^ ^]^ Previous studies using silicon probes in the rodent cortex reported perceptual thresholds that were between 20 and 60 µA (4 and 12 nC per phase), up to 40 weeks.^[^ ^27^, ^28^, ^31^ ^]^ We here report an average perception threshold of 8.3 µA (1.41 nC per phase) using flexible polyimide probes. We did not examine the quality of the perceptual experience of the mice, but it is encouraging that they were able to report V1 stimulation for extended periods, up to 55 weeks, coming close to the entire adult life of a mouse.^[^ ^51^ ^]^ In one animal, we collected multiple measurements of the same electrodes, and perceptual thresholds remained low throughout the experiment, at an average of 3.9 µA (0.66 nC per phase); although, we observed a rise of average detection thresholds at the end of the testing period. Yet, the average threshold current remained well below 10 µA (1.7 nC per phase), below previous studies using silicon probes and well within the capability limits of the electrodes (6.12 nC per phase). Our behavioral results align well with a recent publication by Lycke et al.,^[^ ^26^ ^]^ who used polymer‐based probes for microstimulation in the somatosensory cortex of mice. These authors reported detection thresholds in the range of 0.77 nC per phase and current amplitudes that could be as low as 1 µA, for periods up to 44 weeks. Our findings provide converging evidence in the visual cortex and support the conjecture that polymer probes enable safe stimulation over longer periods, using currents that are lower than those required when using silicon probes (e.g.,^[^ ^27^, ^28^ ^]^).

The lasting efficacy of electrical stimulation implies that a neural interface based on polyimide can remain functional possibly across years because it causes a relatively mild foreign body response. Our histological findings are compatible with this conclusion. Unlike some previous studies,^[^ ^26^, ^52^, ^53^, ^54^ ^]^ we examined the depth profile of tissue reactivity. We observed little response of astrocytes in superficial layers and along the probe shank but a limited reaction deeper in the cortex, at the intersection of the probe trajectory with the white matter. Our results contrast with a previous study which reported less glial scarring at deeper cortical depths,^[^ ^31^ ^]^ but more studies will be needed to understand the factors that determine the tissue reactivity profile across the cortical depth. Our study did not include a systematic comparison between cortical tissue undergoing electrical stimulation and tissue that was not stimulated. However, a previous study in monkeys demonstrated that continuous microstimulation with an amplitude of 100 µA for 4 h per day, 5 days per week during six months did not cause additional tissue damage compared to non‐stimulated electrodes.^[^ ^40^ ^]^ Our stimulation protocol included much lower currents and fewer pulses; and it is therefore likely that the tissue damage was caused by the implant and not by the application of the electrical currents. Our in vitro pulsing data support this view by showing that 10 billion pulses at five times the current used in vivo did not exceed electrochemical safety boundaries.

One previous study demonstrated the absence of glial scar formation upon the implantation of flexible neural probes in the cortex,^[^ ^22^ ^]^ and another study demonstrated that soft microwires elicit a weaker tissue response than stiff microwires.^[^ ^52^ ^]^ Our results are in general agreement with these findings and it seems likely that the cross‐section of the implanted neural probes accounts for the differences between studies.^[^ ^25^, ^55^ ^]^ Specifically, our flexible probes had a thickness of 10 µm, whereas the ones used by Luan et al.,^[^ ^22^ ^]^ who observed even less reactivity, had a thickness of only 1 µm. Indeed, a recent study using a highly flexible mesh probe also reported that a glial response was virtually absent.^[^ ^51^ ^]^ The weak glial responses in our study might therefore be further diminished by decreasing the probe thickness. Future research could examine the influence of probe thickness and width and the influence of the implantation method in a more systematic fashion.

We conclude that thin film polymers with small electrodes provide a promising avenue for sensory prostheses that aim to activate neurons at a high spatial resolution over extended time periods. Substantial work is still needed before the translation of this technology into medical devices that can be safely used in blind people. For example, there is a need to develop methods to safely implant and interconnect large numbers of electrodes in the visual brain; while, covering the entire visual field representation. Substantial effort will also be needed to address all the regulatory aspects as well as wireless implant interconnection and powering. Nevertheless, the present work represents an important step in this direction by demonstrating a long‐lasting, stable, and high‐resolution interface with the visual brain.

## 4 Experimental Section

### Probe Fabrication and Pre‐Characterization

Intracortical polyimide probes were fabricated using tailored lithographic cleanroom processes as described in Boehler et al.^[^ ^21^ ^]^ To reduce the footprint of the implant and improve the tissue integration, multiple metal layers were implemented to incorporate 12 individual electrode sites (area: 15 × 15 µm^2^) into a flexible probe with a width of 39 µm and a thickness of 10 µm (Figure 1A). In total, four metallization layers (Pt, 100 nm) were used for the electrode tracks, which were separated by 2 µm thin polyimide layers and interconnected through vias. SIROF was deposited onto the electrode sites. All SIROF sites were coated with PEDOT/PSS through electrochemical polymerization (see Boehler et al.^[^ ^56^ ^]^) to further reduce the electrode impedance and increase their charge injection capacity. A small hole (diameter 20 µm) was integrated at the tip of the probe to facilitate implantation with a tungsten needle.

The probes were soldered to custom‐made ceramic circuit boards, providing an Omnetics connector for interconnection to the in vitro characterization equipment and to the recording and stimulation devices during the chronic animal experiments. A ground wire (*Ø* 127 µm, Teflon coated stainless steel) connected to a ground‐screw was also implanted on the animal skull. Solder joints were insulated with epoxy (UHU Endfest).

After PEDOT deposition, all probes were characterized by means of cyclic voltammetry (CV) and electrochemical impedance spectroscopy (EIS) using an Autolab 128N Potentiostat (Metrohm) and following the electrode characterization guidelines described in Boehler et al.^[^ ^32^ ^]^

### In Vitro Long‐Term Stability Test

To determine the electrochemically safe charge transfer across the electrode sites and to benchmark their performance under long‐term stimulation conditions, a sub‐set of probes (16 individual electrode sites) was exposed to in vitro pulse‐testing according to the guidelines in Boehler et al.^[^ ^32^ ^]^ These measurements were performed in a two‐electrode configuration and using 0.01 m phosphate buffered saline (PBS) as electrolyte. Stimulation was done with a biphasic, cathodic first current pulse at a pulse duration of 170 µs per phase and with an inter‐pulse delay of 65 µs. Pulses were repeated at a frequency of 1 kHz to allow testing under exaggerated stress conditions, and the voltage over the individual electrode sites was recorded.

Although resilience to pulsing was primarily determined by the electrode materials, it may also depend on the stimulator type, especially at high pulsing frequencies, which increase the probability of the accumulation of charge. Therefore, the test was repeated with a Blackrock CereStim R96 stimulator to demonstrate that long term stability during charge injection could be replicated with a second instrument (Figure S2, Supporting Information).

To determine the electrochemically safe maximal charge transfer (*Q*_max_), the current amplitude was increased until the recorded voltage drop across the electrode reached the limits known as the water window.^[^ ^32^ ^]^ The corresponding stimulation current was used to calculate the *Q*_max_ value.

Continuous long‐term stimulation was performed in three stimulation groups, applying either 75% of *Q*_max_ (group 1), 50% of *Q*_max_ (group 2), or no charge (group 3) to the respective electrode sites (*n* = 8 per group) for a total number of 10 billion pulses; while, monitoring the voltage drop across the electrode. The electrochemical characteristics before and after these pulses were compared to evaluate the stability of the electrodes, and SEM was used to examine the morphology of the coating. After an initial increase in polarization, the electrodes reached a stable potential for the two current levels, suggesting that the initial polarization was caused by variation in the environment (e.g., change in ambient temperature or salt concentration) and not by a change in the electrode material.

### Mice and Surgical Procedures

Seven C57BL/6J mice (three male, four female), which were implanted at 12–14 weeks of age, were used. Five of these mice were part of the stimulation experiment; two mice were only used for histological purposes. The animals were solitarily housed on a reversed day/night cycle for the duration of the experiment. The cages were enriched with extra bedding, a running wheel, and a tunnel. The study protocol (AVD‐801002016631) was approved by the CCD (Central Commissie Dierproeven) and the ethical committee of the Royal Netherlands Academy of Arts and Sciences.

Before the surgery, the mice were anesthetized with 3–4% isoflurane in an induction box and anesthesia was maintained during surgery with 1.5–2% isoflurane in an oxygen‐enriched air (50% air and 50% O_2_) mixture. The depth of anesthesia was continuously monitored based on pinch‐reflexes and breathing rate and the isoflurane intake was adjusted accordingly. As a general analgesic, an intraperitoneal injection of 5 mg kg^−1^ Metacam was used. A heating pad was used to maintain a body temperature between 36.5 °C and 37.5 °C. The animals were head‐fixed in a stereotaxic frame and an eye‐cover was applied with Bepanthen cream to prevent dehydration of the eyes. The hair on the head of the animal was shaved off and the skin was cleaned. Before making an incision, a small amount of Xylocaine sprayed on the skin was applied for additional local analgesia. The skin was then cut to expose the area of the skull above the visual cortex and an extended area posterior to lambda. The skull was cleaned, and a dental primer was applied to enable secure fixation of cement to the skull.

Prior to surgery, the MANTA probe was glued to a tungsten wire shuttle device with bio‐dissolvable adhesive polyethylene glycol (PEG), and the probe was mounted in the stereotaxic frame. A small, circular craniotomy was drilled over the left or right visual cortex. Insertion sites were chosen over V1 at 3.5 mm posterior to Bregma on the AP‐axis and 2.5 mm sagittal on the ML‐axis. The shuttle device with the probe was positioned over the craniotomy and inserted into the cortex until it reached a depth of 900 µm. The authors waited 3 min to allow the PEG to dissolve; while, continuously applying saline solution on the tungsten wire outside of the cortex to separate the probe from the shuttle device. Afterward, the tungsten wire was slowly retracted; while, leaving the probe behind, which was fixed to the skull by sealing the craniotomy with dental cement. A screw was attached into the skull over the cerebellum and the probe's ground wire was wrapped around it several times. At the end of the surgery, the probe connector and head‐bar were mounted on top of the skull with dental cement.

### Intracortical Microstimulation

Microstimulation was delivered with a Blackrock CereStim R96 stimulator. Stimulation pulses were biphasic, cathodic‐leading, and monopolar. The duration of the two phases was 170 µs, and the interphase interval was 60 µs. Pulse trains were applied with a frequency of 300 Hz. The number of pulses was 60 in the training phase (total train duration of 240 ms), and 30 during the threshold measurements (total pulse train duration of 120 ms). The maximum amplitude applied via microstimulation was 25 µA, resulting in a maximum charge injection per phase of 4.25 nC per phase. The maximum current used in this study was well below the maximal currents for safe stimulation, as had been outlined by Shannon.^[^ ^57^ ^]^

### Behavioral Training

During behavioral training and testing, the mice were under a controlled fluid uptake regime, with a minimal intake of 0.025 mL g^−1^. The animals were trained to lick when an electrode was stimulated. The training consisted of two phases. In the first, passive training phase water dispensation and stimulation were presented simultaneously. In the second, active training phase mice had to perform a licking response to the stimulation to receive the water reward. Stimulation amplitude during the training phases was kept constant at 25 µA. The paradigm consisted of a randomized presentation of stimulation and non‐stimulation trials. Stimulation trials started with a single presentation of the stimulation pulse train at an amplitude of 25 µA, followed by a 0.5 s response window and an inter‐trial interval of 3–5 s. A 3−6 s no‐lick period was introduced at the end of each trial to discourage constant licking behavior. For non‐stimulation trials, a false alarm timeout of 3–10 s occurred if animals licked without the presentation of stimulation. The signal detection theory was used to compute the d‐prime based on the proportions of hits and false alarms (i.e., Z[*P*_Hit_] − Z[*P*_False alarm_]). When the d‐prime was higher than 1.5, the mice were considered proficient enough to continue with psychometric testing of detection thresholds.

### Measurement of Current Thresholds

Psychophysical experiments were run to determine the perceptual thresholds, using different current amplitudes in a randomized order with 10–20 repetitions per amplitude. 30% catch trials were included without stimulation to determine the false alarm rate. The psignifit toolbox (version 2.5.6), which implements the maximum‐likelihood method described by Wichmann & Hill,^[^ ^58^ ^]^ was used to fit the psychometric functions. Thresholds were defined as the minimal current amplitude necessary for 60% correct responses. Thresholds were presented as mean ± SD unless stated otherwise. A server crash caused the loss of several computer files, but the accuracy of the mice and number of trials for each stimulation amplitude were stored in another computer; so that, the data of all test sessions could be included.

### Histological Analysis

The mice were deeply anesthetized by injecting 0.3 mL of Pentobarbital (60 mg mL^−1^) intraperitoneally, and the mice were perfused following a three‐step protocol, starting with PBS, followed by 4% paraformaldehyde (PFA) in PBS, and concluding with 15% sucrose in PBS. The implants were removed during brain extraction from the skull and stored for further inspection. For histological purposes, the brains of two mice who were implanted with the electrode array for 26 weeks but did not receive stimulation were analyzed. To identify the insertion‐point of the probes in the brain, it was immersed into a solution of 10% Evans’ Blue (50 mg 100 mL^−1^) in PBS for 20 min. Evans’ Blue stains albumin and allowed visualization of sites with blood leakage that occurred during the insertion of the probes. Following the identification of the insertion site, a region of interest (ROI) was identified by medio‐lateral markings on the surface of the brain, located ≈1 mm anterior and posterior from the insertion point. 50 µm‐thick coronal sections were cut within this ROI, for further analysis using a Leica CM3050 cryostat. The brain sections were stored floating in a 50% glycerol in PBS solution. The analysis was further focused by identifying sections presenting insertion lesions (visible in an average of three slices). To examine neurons and astrocytes, the primary antibodies NeuN (mouse anti NeuN, Invitrogen, MA5‐33103) and GFAP (goat anti GFAP, Sigma–Aldrich, SAB2500462) were used. Alexa fluor conjugated secondary antibodies were used with GFAP (Alexa 594 donkey anti‐goat, Jackson ImmunoResearch, 705‐585‐147) and Cyonine with NeuN (Cy3 donkey anti‐mouse, Jackson ImmunoResearch, 715‐165‐150). The sections were mounted on glass slides and imaged on a Leica SP8 confocal laser scanning microscope system.

The GFAP and NeuN signal were examined by quantifying the signal intensity of each pixel around the probe lesion. For this, the images of two animals were downsampled to a resolution of 50 × 63 and centered to the site of the lesions. Three regions of interest (ROI) were identified for analysis: 1) superficial layers, 2) along the shaft of the probe, and 3) at the tip of the probe. In each ROI, five horizontal lines were selected, one pixel apart, per animal (i.e., ten lines per ROI). The signal intensity was defined as the average of these ten lines for each ROI. Both in superficial layers and at the shaft of the probe, a fluorescent light artefact was present in the NeuN signal. In the superficial layers, the artefact was small enough to bridge over it. At the shaft of the probe, the data points of the artefact were removed.

### FIB‐SEM Analysis

To prepare the explanted probes for imaging, the skull and the dental acrylic fixing the connector were trimmed to create an optical access to the probe. Images were first collected with a regular stereomicroscope (Zeiss Discovery V8). Then, the explant was coated with a thin layer of gold (7 nm, CCU‐010, Safematic) and transferred to an SEM (Helios 5, Thermo Fisher Scientific) for high‐resolution imaging of electrode sites.

## Conflict of Interest

P.R.R. is cofounder and shareholder of Phosphoenix (Netherlands), which is a neurotechnology start‐up.

## Supporting information

Supporting Information

## Data Availability

The data that support the findings of this study are available from the corresponding author upon reasonable request.
