# Supplementary material for: Flexible Polymer Electrodes for Stable Prosthetic Visual Perception in Mice
Source: Adv Healthc Mater. 2024 Mar 3;13(15):2304169. doi: 10.1002/adhm.202304169 (PMC11468866; doi:10.1002/adhm.202304169)
Supplement: Supplementary file 1 — Supporting Information [file ADHM-13-2304169-s001.pdf]

ADVANCED HEALTHCARE MATERIALS

# Supporting Information

for *Adv. Healthcare Mater.*, DOI 10.1002/adhm.202304169

Flexible Polymer Electrodes for Stable Prosthetic Visual Perception in Mice

*Corinne Orlemann, Christian Boehler, Roxana N. Kooijmans, Bingshuo Li, Maria Asplund* and Pieter R. Roelfsema**

## Supplementary Information

*Detailed analysis of failure modes*

We analyzed the behavioral data of probes that had been implanted in 5 mice, with one probe per mouse. For mouse 1 we collected the perceptual thresholds of 7 electrodes. The average threshold was 3.3 ± 0.9 μA (0.6 ± 0.15 nC/phase; range = 2.5 to 5.1 μA). This initial threshold testing of the 7 electrodes occurred between 12 and 18 weeks of implantation. Stimulation of the remaining 4 electrodes yielded no behavioral response. These electrodes had a high impedance (> 20 MOhm) and were presumably not connected on the ceramic PCB. We repeated measurements of the 7 active electrodes at 5 additional timepoints (up to 55 weeks) to measure threshold changes over time (see Results, Figure 2D, and Table S2). Data collection was stopped because the mouse reached a humane endpoint due to old age. The average threshold for 5 electrodes in mouse 2 was 5.9 ± 3.7 μA (1 ± 0.6 nC/phase; range = 1.8 to 11 μA). These thresholds were collected between 6 and 13 weeks of implantation. For the remaining 6 electrodes we were not able to determine a threshold, because stimulation via this probe did not elicit behavioral responses after 14 weeks. For mouse 3 we calculated an average perceptual threshold of 10.4 ± 3.4 μA across 7 electrodes (1.8 ± 0.6 nC/phase; range = 6.6 to 15.3 μA). These thresholds were collected between 12 and 24 weeks of implantation. We were not able to repeat threshold measurements of these same electrodes at a later timepoint due to a technical issue, but the mouse performed well on the go/no-go detection task for up to 37 weeks. For 2 of the remaining electrodes, we measured a high impedance (> 20 MOhm) for the entire duration of the experiment. The impedance of two additional electrodes remained in normal ranges during the experiment but stimulation did not lead to a behavioral response. For mouse 4 perceptual thresholds of 3 electrodes yielded an average of 18.2 ± 3 μA (3.1 ± 0.5 nC/phase; range = 14.8 to 20.2 μA). These thresholds were collected between 7 and 13 weeks of implantation. We were not able to determine thresholds of further electrodes as the connector on the animal's head broke off during one session after 15 weeks. For mouse 5, behavioral training responses were recorded for 4 electrodes, but we only obtained the perceptual threshold of 1 electrode of 10.6 μA (1.8 nC/phase). This threshold was collected 14 weeks after implantation. Later problems with the connector occurred, and stimulation did not elicit any further behavioral responses. Table S1 shows the initial perceptual thresholds and failure modes of stimulation of each electrode in all probes.

## Supplementary Tables

| | | | Threshold / Failure Mode | | |
|---|---|---|---|---|---|
| Electrode | mouse 1 | mouse 2 | mouse 3 | mouse 4 | mouse 5 |
| | μA \| nC/phase | μA \| nC/phase | μA \| nC/phase | μA \| nC/phase | μA \| nC/phase |
| 1 | HI | 1.8 \| 0.31 | HI | 19.7 \| 3.35 | - |
| 2 | HI | 4.3 \| 0.73 | HI | 20.2 \| 3.43 | - |
| 3 | HI | 11 \| 1.87 | 9.9 \| 1.68 | 14.8 \| 2.52 | - |
| 4 | 2.5 \| 0.43 | - | 9.8 \| 1.67 | BC | - |
| 5 | 2.9 \| 0.49 | - | 7.1 \| 1.21 | BC | 10.6 \| 1.8 |
| 6 | 3.5 \| 0.6 | - | 9 \| 1.53 | BC | - |
| 7 | HI | - | 6.6 \| 1.12 | BC | - |
| 8 | 5.1 \| 0.87 | 8.3 \| 1.41 | 14.8 \| 2.52 | BC | - |
| 9 | 2.7 \| 0.46 | 4 \| 0.68 | 15.3 \| 2.6 | BC | - |
| 10 | 3.2 \| 0.54 | - | NR | BC | - |
| 11 | 3.1 \| 0.53 | - | NR | BC | - |
| Duration | 55 weeks | 14 weeks | 37 weeks | 15 weeks | 30 weeks |

**Table S1 Detection thresholds and failure modes.** Detection thresholds and failure modes of each electrode for all five probes. The first number in each cell is the threshold in μA and the second number shows nC/phase. For 6 out of the 23 electrodes we could no longer elicit perceptions when time had progressed. HI, Electrode with a high impedance (>20 MOhm). -, Electrode not tested. NR, No behavioral response was elicited via stimulation. BC, The connector broke off so that the electrode could not be tested. Duration refers to the maximum duration at which stimulation of electrodes elicited a behavioral response. For mouse 1, only the thresholds of the initial testing are represented. No stimulation failure occurred in 3 out of the 5 probes and the experiment concluded because the animal reached a humane endpoint. Stimulation failures of the other 2 probes occurred after 14 and 30 weeks of implantation.

| Electrode | T1: 16 w | T2: 24 w | T3: 29 w | T4: 34 w | T5: 49 w | T6: 55 w |
|---|---|---|---|---|---|---|

| | µA \| nC/phase | µA \| nC/phase | µA \| nC/phase | µA \| nC/phase | µA \| nC/phase | µA \| nC/phase |
|---|---|---|---|---|---|---|
| 1 | X | X | X | X | X | X |
| 2 | X | X | X | X | X | X |
| 3 | X | X | X | X | X | X |
| 4 | 2.5 \| 0.43 | 2.5 \| 0.43 | 1.9 \| 0.32 | 1.5 \| 0.26 | 3.6 \| 0.61 | 6.2 \| 1.05 |
| 5 | 2.9 \| 0.49 | 2.1 \| 0.36 | 2.6 \| 0.44 | 2.6 \| 0.44 | 3.3 \| 0.56 | 7.8 \| 1.33 |
| 6 | 3.5 \| 0.6 | 1.1 \| 0.19 | 2.2 \| 0.37 | 2.5 \| 0.43 | 5.8 \| 0.99 | 5.8 \| 0.99 |
| 7 | X | X | X | X | X | X |
| 8 | 5.1 \| 0.87 | 4.3 \| 0.73 | 3.4 \| 0.68 | 3 \| 0.51 | 5.6 \| 0.95 | 5.7 \| 0.97 |
| 9 | 2.7 \| 0.46 | 3.6 \| 0.61 | 3.7\| 0.58 | 2 \| 0.34 | 6.1 \| 1.04 | 6.3 \| 1.07 |
| 10 | 3.2 \| 0.54 | 2.6 \| 0.44 | 4 \| 0.68 | 2.5 \| 0.43 | 7.5 \| 1.28 | 6.5 \| 1.11 |
| 11 | 3.1 \| 0.53 | 3 \| 0.51 | 4.8 \| 0.82 | 2.7 \| 0.46 | 6.1 \| 1.04 | 4.5 \| 0.77 |

**Table S2 Detection thresholds of mouse 1 across 55 weeks**

The first number in each cell is the threshold in µA and the second number shows nC/phase. Electrodes for which no threshold was determined are marked with an X.

**Supplementary Figures**

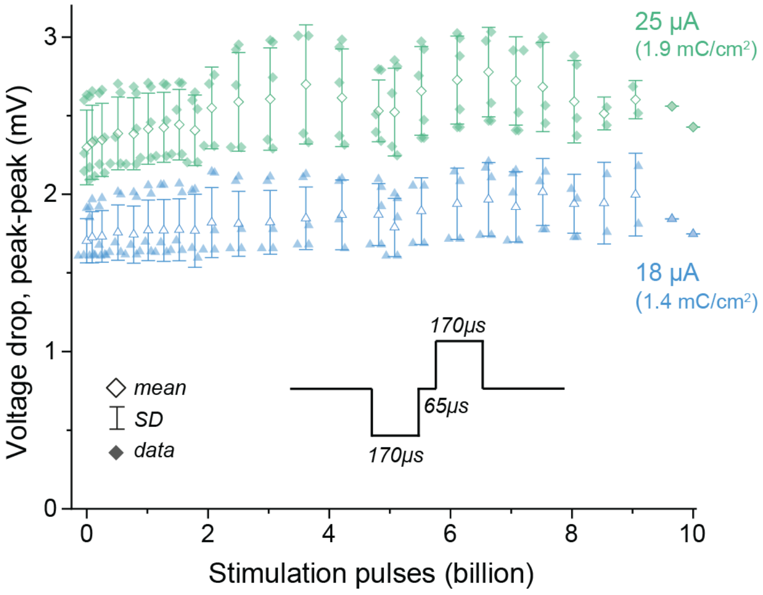


**Supplementary Figure 1: In-vitro long-term stimulation data.** Full data-set showing the voltage drop over the individual electrode sites during biphasic pulsing at 18 µA (blue triangles) and 25 µA (green diamonds) during 10 billion stimulation pulses applied during a period of 16 weeks. Hollow symbols represent mean values (standard deviation as error bars) and solid symbols mark individual datapoints. Over the entire stimulation period, the voltage drop increased from an average of 1.7 ± 0.1 V to 1.9 ± 0.2 V (14%), and the voltage drop at 25µA increased from 2.3 ± 0.2 V to 2.6 ± 0.3 V (13%).

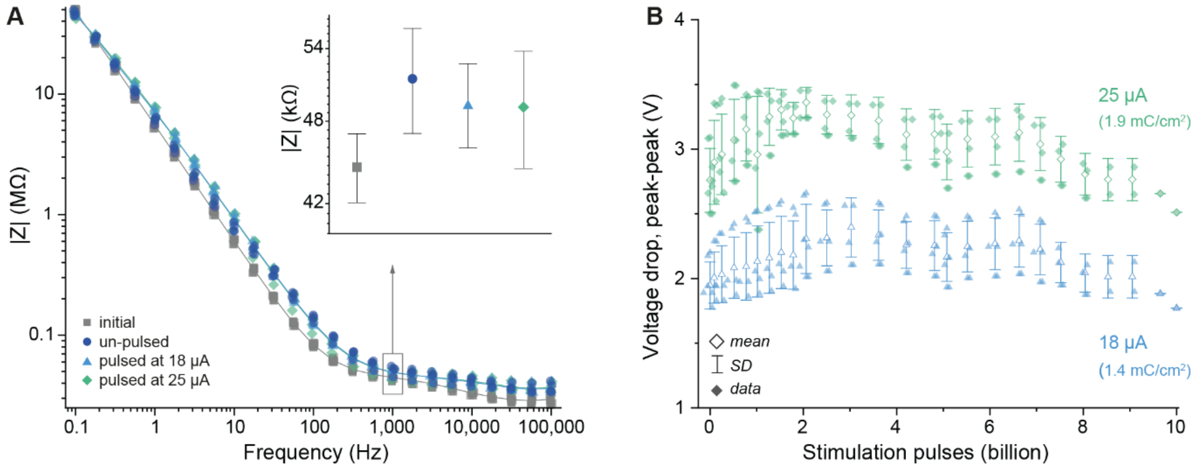


**Supplementary Figure 2: In-vitro pulsing data with a different device for electrical stimulation.** Long-term pulsing of electrode sites at various current amplitudes was repeated with a Blackrock Cerestim R96 stimulator to evaluate the effect of residual charge built-up on the electrodes with a different charge compensation circuitry (the results in Figure. 1B were obtained with a Plexon stimulator). **A.** Impedance comparison for electrodes prior to pulsing

(grey squares), non-pulsed control electrodes (blue circles) and electrodes exposed to 10 billion stimulation pulses at 18 μA (blue triangles) and 25μA (green diamonds). The inset shows the mean ± SD for the individual groups at a frequency of 1 kHz. **B.** Voltage drop in response to the biphasic stimulation, recorded over a period of 16 weeks (10 billion pulses). Hollow symbols represent mean values with standard deviation as error bars, solid symbols mark individual datapoints.
